# Declining HIV-1 prevalence and incidence among Police Officers – a potential cohort for HIV vaccine trials, in Dar es Salaam, Tanzania

**DOI:** 10.1186/1471-2458-13-722

**Published:** 2013-08-06

**Authors:** Patricia J Munseri, Muhammad Bakari, Mohamed Janabi, Eric Aris, Said Aboud, Bo Hejdeman, Eric Sandstrom

**Affiliations:** 1 Department of Internal Medicine, School of Medicine, Muhimbili University of Health and Allied Sciences, Dar es Salaam, Box 65001, Tanzania; 2 Venhalsan Sodersjukhuset ,Karolinska Institutet, Stockholm, Sweden; 3 Department of Internal Medicine, Muhimbili National Hospital, Dar es Salaam, Tanzania; 4 Department of Microbiology and Immunology, Muhimbili University of Health and Allied Sciences, Dar es Salaam, Tanzania

**Keywords:** HIV, Incidence, Prevalence, Cohort for vaccine trials, Tanzania

## Abstract

**Background:**

A safe effective and affordable HIV vaccine is the most cost effective way to prevent HIV infection worldwide. Current studies of HIV prevalence and incidence are needed to determine potentially suitable cohorts for vaccine studies. The prevalence and incidence of HIV-1 infection among the police in Dar es Salaam in 1996 were 13.8% and 19.6/1000 PYAR respectively. This study aimed at determining the current prevalence and incidence of HIV in a police cohort 10 years after a similar study was conducted.

**Methods:**

Police officers in Dar es Salaam, Tanzania were prospectively enrolled into the study from 2005 and followed-up in an incidence study three years later. HIV infection was determined by two sequential enzyme linked immunosorbent assays (ELISAs) in the prevalence study and discordant results between two ELISAs were resolved by a Western blot assay. Rapid HIV assays (SD Bioline and Determine) were used for the incidence study.

**Results:**

A total of 1,240 police participated in the HIV prevalence study from August 2005 to November 2008. Of these, 1101 joined the study from August 2005-September 2007 and an additional 139 were recruited between October 2007 to November 2008 while conducting the incidence study. A total of 726 (70%) out of the 1043 eligible police participated in the incidence study.

The overall HIV-1 prevalence was 65/1240 (5.2%). Females had a non-statistically significant higher prevalence of HIV infection compared to males 19/253, (7.5%) vs. 46/987 (4.7%) respectively (p = 0.07). The overall incidence of HIV-1 was 8.4 per 1000 PYAR (95% CI 4.68-14.03), and by gender was 8.8 and 6.9 per 1000 PYAR, among males and females respectively, (p = 0.82).

**Conclusions:**

The HIV prevalence and incidence among the studied police has declined over the past 10 years, and therefore this cohort is better suited for phase I/II HIV vaccine studies than for efficacy trials.

## Background

The overwhelming burden of HIV in sub-Saharan Africa is of major concern and has claimed many lives in the region [1].

Despite multiple preventive measures in place for the control of HIV and AIDS, the disease still remains to be of public health importance. According to the UNAIDS report of 2011 there were 2.7 million new HIV infections worldwide in 2010 [2]. While in Tanzania the HIV prevalence was noted to be 6% [3].

Although the availability of highly active antiretroviral therapy (HAART) has dramatically improved the quality of life and life expectancy of people living with HIV and AIDS, the ultimate control of this pandemic probably depends upon the availability of a safe, effective and affordable HIV vaccine.

Several vaccine candidates at different phases of trials, including phase III trials have been tested in humans in different continents and have been found to be safe and to elicit immune response, including the recently concluded phase III study conducted in Thailand that revealed a 31% efficacy [4].

Participation of sub-Saharan Africa in HIV vaccine trials is crucial, since this is the region that bears the major brunt of HIV infection and would be a potential region to benefit most from a vaccine [5].

There is a need to prepare cohorts of volunteers for HIV vaccine studies and moreover there is a need to assess the suitability of these cohorts for such studies.

For phase I/IIa HIV vaccine studies the required cohort is supposed to be of low risk for HIV infection. For efficacy studies where there is need to determine the efficacy of the vaccine candidate in question in the midst of other non vaccine interventions the cohort to be studied is supposed to be at a relatively high risk for HIV infection [6].

The police cohort in Dar es Salaam, Tanzania was established in 1994 in preparation for phase I/II HIV vaccine trials in Dar-es Salaam. The initial HIV prevalence of 13.8% in this cohort was similar to the general population prevalence for that year, while the incidence was noted to be 19.9/1000 PYAR [7]. This is in the realm of incidence where HIV vaccine efficacy trials are feasible.

A subgroup of these police officers, with low risk for HIV infection, participated in a recently conducted phase I/II HIV vaccine trial (HIVIS 03) to ascertain the safety and immunogenicity of the candidate HIV-1 DNA-MVA vaccines [8].

During recruitment in the incidence study in 1994–1996, study subjects were given HIV prevention educational sessions [7]. Furthermore a core group of more than 300 police officers was established and maintained with a focus to educate them more about HIV infection and prevent transmission of HIV among members in the police force.

HIV incidence in cohorts has been shown to decline over time in many African countries including Tanzania [1]. Our research group has maintained contacts with the police force as a whole with regards to HIV education and prevention as well as participation of the police, among other cohorts, in phase I/II HIV vaccine trials since 1994.

The aim of this study was therefore to determine the current HIV prevalence and incidence among the police in Dar es Salaam. This study is conducted 10 years following the initial HIV prevalence and incidence study so as to determine if the police force is still a suitable cohort for HIV vaccine efficacy studies.

## Methods

### Setting

The study was conducted at all the 32 police stations in Dar es Salaam, Tanzania, which were located in all the three municipalities of urban and peri-urban Dar es Salaam.

### Design

Prospective Cohort study.

### Study period

Recruitment began in August 2005 and follow up was complete in November 2008.

### Study population

Were police men and women aged 18 years or above from Dar es Salaam Tanzania. They were prospectively recruited into the study following educational sessions on HIV and AIDS that were done in their respective stations. Physicians conducted the educational sessions with the help of collaborators from the Police force that included Doctors and Nurses from the police health unit on a weekly basis. After each educational session, the police were invited to participate in the study, prior to participation in the study an informed consent was obtained from each of the police.

Trained nurse counselors performed pre and post-test HIV counseling to all study subjects. The nurse counselors also filled in a standardized questionnaire that included socio-demographic data, social behavioral questions on HIV risk and questions on HIV testing. After the interview sessions the study participants were requested to donate blood for HIV testing.

Following HIV testing in the Microbiology and Immunology laboratory at the Muhimbili University of Health and Allied Sciences, the HIV results were communicated to the respective study subject at their respective stations a week later whereby post-test counseling was performed by a nurse counselor.

### Follow up

For the incidence study the study staff visited the stations from October 2007 to November 2008 and requested all those who participated in the prevalence study in 2005–2008 to re-test for HIV. Only participants who were not HIV infected in the prevalence study were tested for HIV in the incidence study.

### Blood sample collection

Two milliliters of venous blood was collected for HIV testing at the Department of Microbiology and Immunology, Muhimbili University of Health and Allied Sciences (MUHAS).

### HIV testing

For the HIV prevalence study: HIV serostatus was determined using the Murex antigen/antibody combination (Abbott, UK) enzyme linked immunosorbent assays (ELISA) followed by testing of reactive sample on the Enzygnost anti-HIV-1/HIV-2 plus (Behring, Marbug, Germany) assay as previously used [9]. Discordant results between two ELISAs were confirmed using Inno-Lia immunoblot assay (Innogenetics, Belgium).

For the HIV Incidence study: HIV serostatus was determined using the National rapid HIV testing algorithm where initial testing was performed using SD Bioline (Standard Diagnostic Inc, Korea) followed by confirmation of reactive samples on Determine (Inverness Medical, Japan) as used previously [10]. Sera reactive on both tests were considered as positive. Uni-Gold assay (Trinity, UK) was used as a tiebreaker. The change in testing methodology was determined by the fact that the study participants did not want to wait for a week for their HIV test results. Furthermore it was noted that there were several voluntary and counseling testing sites that offered HIV testing and results on the same day.

### Social characteristics

Information on social behavioral characteristics was collected on a subgroup of individuals who participated in the prevalence study in 2005. This subgroup only included the police who were recruited consecutively in the study for the first six months. Thereafter this information was not collected as it was noted that this consumed a lot of time as the police needed to attend to their duties.

### Ethical clearance

The study received ethical clearance from the Muhimbili University of Health and Allied Sciences ethics committee. Written informed consent was obtained from each study participant prior to the inclusion in the study. Informed consent was obtained from each police who participated and there was no coercion from the higher authorities in the Police Force.

### Treatment

All study subjects who were found to be HIV-infected were referred to the Muhimbili National Hospital, HIV care and treatment clinic and were offered standard of care according to the Tanzania National Guidelines for Care and Treatment of HIV-infected patients.

### Data analysis

Data were analyzed using SPSS version 18 software (SPSS, Chicago, IL, US). 95% Confidence intervals were computed using Open Epi version 2 that is available on line in the following link (http://www.openepi.com/OE2.3/PersonTime1/PersonTime1.htm) the test was used to compare proportions and incidence rates between the sexes. Two by two tables were used to compare social demographic variables between males and females. Comparisons were made for the social demographic variables by gender as stratified by HIV status. Comparison of proportions was made using the Chi square test. HIV incidence was estimated from the time the first HIV test was negative to the time the test was positive. Since participants had different follow up times. The incidence rates were calculated as the number of new HIV infections divided by the person time years of observation for each study participant. Relative risk was used to compare the HIV incidence by gender and statistical significance was judged using a 95% confidence interval. A double sided p value of <0.05 was considered to be statistically significant.

## Results

### Socio-demographic characteristics

A total of 1,367 police from the 32 stations in Dar-es Salaam were provided with education on HIV by the study team. Of these 1,244 (91%) consented for a blood withdrawal for HIV testing: 991/1244 (80%) were male.

There was no significant difference between females and males with respect to agreeing to a blood withdrawal and subsequent HIV testing 253/282 (90%) vs 991/1085 (91%), respectively (p = 0.64). When it came to actual testing 1240 (99.7%) were tested for HIV, with only 4 (0.3%) participants who had consented not showing up for the blood test.

The demographic characteristics of the study population are shown in Table 1. Only a subset of the study population were asked questions on social risk behaviour for practical reasons, due to a tight schedule at the working place that left minimal time for the study participants to fill in the questionnaire. The sub group comprised of 435 police, 88 females and 347 males from the police who were recruited into the study during the first six months of the study.

**Table 1:** Socio-demographic and behavioral characteristics of the study participants who participated in the first round of the prevalence study

**Characteristic**	**Male**	**Female**	**P value**
Median age (range)	37 (18–61)	33 (18–57)	<0.001
Mean Age (SD)	36 (±9.2)	34 (±9.5)	
Marital Status (n=435)			
Never married	88 (25.4%)	40 (45.5%)	<0.001
Married	259 (74.6%)	48 (55.5%)	
Level of education (n=433)			
Primary	138 (40.0%)	38 (43.2%)	
Secondary	189 (54.8%)	47 (53.4%)	0.751
College/university	18 (5.2%)	3 (3.4%)	
Condom use last sexual encounter (n=307)			
Yes	28 (10.9%)	9 (17.6%)	0.163
No	228 (89.1%)	42 (82.4%)	
Regular sexual partner Wife/Husband (n=304)			
Yes	237 (93.3%)	35 (70.0%)	<0.001
No	17 (6.7%)	15 (30.0%)	
Regular sexual partner someone else (n=226)			
Yes	105 (59.7%)	29 (58.0%)	
No	71 (40.3%)	21 (42.0%)	0.871
Condom use during extramarital sex (n = 21)			
Yes	12 (70.6%)	4 (100%)	0.50
No	5 (29.4%)	0 (0%)	

The females who participated in the study were much younger compared to males with a median age of 33 years compared to 37 years respectively p < 0.001. Likewise a significantly higher proportion of females (45.5%) were not married compared to males (25.4%) p<0.001.

### Participant recruitment

**Figure 1:**
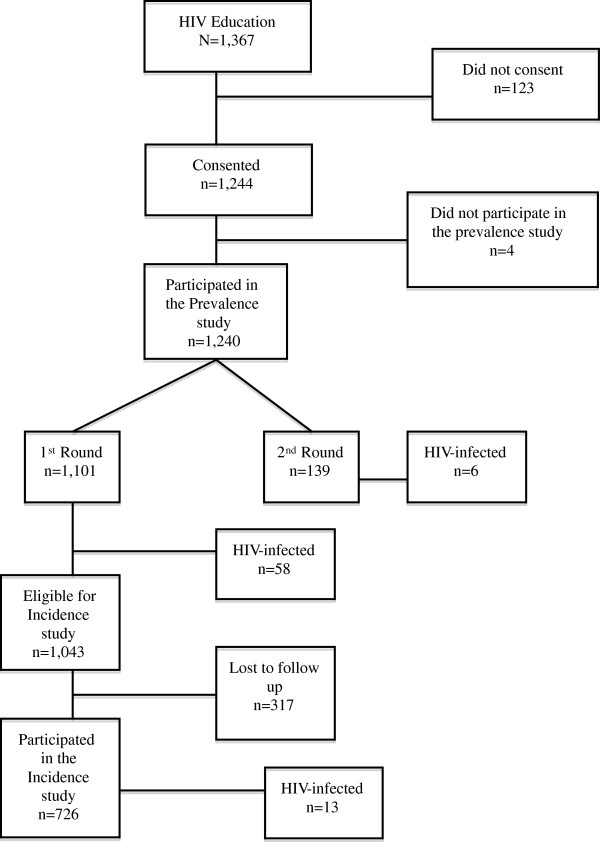
Consort diagram.

Recruitment of study participants and the flow is shown in Figure 1. Of the 1,240 who tested for HIV 1,101 (89%%) were seen in the first round from August 2005 to August, 2007. The remaining 139 were tested for HIV prevalence during the second round while conducting the incidence study that took place between October 2007 and November 2008.

A total of 726 out of the 1,043 (70%) eligible police were seen for a second time for the incidence study between October 2007- November 2008. The remaining 317 (30%) police were lost to follow up. Of these, 65 (21%) were female, and 252 (79%) were male. We were able to obtain reasons for not re-testing in 213/317 (67%). The reasons included: being transferred permanently 63 (30%); being away on special assignment during the study period 53 (25%), retired in 21 (10%), being on vacation 19 (9%); being on study leave 17 (8%), withdrawn consent 16 (7%), terminated from the force 12 (6%); death 5 (2%), hospitalized 3 (1%), resigned 2 (1%), and travelled 2 (1%).

#### HIV prevalence

The overall prevalence of HIV-1 infection was 65/1240 (5.2%) with a statistically non-significant higher prevalence among females 19/253 (7.5%) compared to males 46/987 (4.7%) p = 0.07. The HIV prevalence during the first round was 58/1101 (5.3%) and in the second round was 6/139 (4.3%).

Socio-demographic characteristics related to HIV serostatus are shown in Table 2. HIV infection was common among the older police officers for both males and females, however this difference by gender was not statistically significant. Further, men who refused to test were younger compared to the men who tested for HIV.

**Table 2:** HIV Prevalence by socio- demographic and behavioral characteristics

	**All**	**Males**	**Females**	**Total**
Median age (years range)				
HIV-infected	39 (22–51)	39 (26–51)	38 (22–49)	
HIV-uninfected	36 (18–61)	37 (19–61)	34 (18–56)	0.07
Refused to test	30 (18–57)	30 (18–35)	46 (25–57)	
Marital Status				
(n = 435)				
Never married				
HIV-infected	3 (2.3%)	2 (2.3%)	1 (2.5%)	
HIV-uninfected	125 (97.7%)	86 (97.7%)	39 (97.5)	
				0.03*
Married				
HIV-infected	24 (7.8%)	20 (7.7%)	4 (8.3%)	
HIV-uninfected	283 (92.2%)	239 (92.3%)	44 (91.7%)	
Level of education				
n=433				
Primary				
HIV-infected	16 (9.1%)	13 (9.4%)	3 (7.9%)	
HIV-uninfected	160 (90.9%)	125 (90.6%)	35 (92.1%)	
Secondary				0.06**
HIV-infected	11 (4.7%)	9 (4.8%)	2 (4.1%)	
HIV-uninfected	225 (95.3%)	180 (95.2%)	45 (95.7%)	
College/university				
HIV-infected	0	0	0	
HIV-uninfected	21	18	3	
Condom use last sexual encounter				
n=307				
Yes				
HIV-infected	3 (8.1%)	1 (3.6%)	2 (22.2%)	
HIV-uninfected	34 (91.9%)	27 (96.4)	7 (77.8%)	
				0.76***
No				
HIV-infected	21 (7.8%)	18 (7.9%)	3 (7.1%)	
HIV-uninfected	249 (92.2%)	210 (92.1%)	39 (92.9%)	
Regular sexual partner Wife/Husband				
n=304				
Yes				
HIV-infected	22 (8.1%)	19 (98.0%)	3 (8.6%)	
HIV-uninfected	250 (91.9%)	218 (92.0%)	32 (91.4%)	
				0.93****
No				
HIV-infected	2 (6.3%)	1 (5.9%)	1 (6.7%)	
HIV-uninfected	30 (93.7%)	16 (94.1%)	14 (93.3%)	
Regular sexual partner someone else				
n=226				
Yes				
HIV-infected	6 (4.5%)	6 (5.7%)	0	
HIV-uninfected	128 (95.5%)	99 (94.3%)	29	
				0.95
No				
HIV-infected	5 (5.4%)	3 (4.2%)	2 (9.5%)	
HIV-uninfected	87 (94.6%)	68 (95.8%)	19 (90.5%)	

* p values comparison of HIV –infection among the married to the never married.

** p values comparison of HIV-infection in primary vs secondary education.

*** p values comparison of HIV-infection among the condom users and non condom users.

**** p values comparison of HIV-infection with respect for regular sexual partners and irregular sexual partners.

The HIV-1 prevalence was higher among married compared to never married police, (7.8% vs 2.3%, p = 0.03). HIV infection seemed to be more prevalent among those with lower levels of education compared to those with higher level of education; 9.1% vs 4.7% p = 0.06.

### HIV incidence

Of the 1,043 police who were HIV-negative at recruitment during the prevalence study in 2005, 726 (70%) were followed up until 2008 and generated a total of 1,538 person time years of observation (PYAR). Among the 726 police who were followed up 13 police seroconverted. The overall crude HIV-1 incidence was thus 13/726 (1.79%). The HIV incidence by gender and age are shown in Table 3. The crude HIV-1 incidence by gender was 1.4% (2/143) for females and 1.9% (11/583) for males, p = 0.98. The incidence rate of HIV-1 was 13/1538 (0.85%) or 8.5 per 1000 PYAR (95% CI 4.68-14.03). By gender this was 0.88% (8.8/1000 PYAR) among males, and 0.69% (6.9/1000 PYAR) for females with a relative risk of 0.8 (95% CI 0.31-8.39), p = 0.82 for females compared to males.

**Table 3:** Incidence of HIV-1 per 1000 PYAR by age and sex

**Age groups (years)**	**Total number of males**	**Males PYAR**	**HIV Cases**	**Incidence**	**Total number of females**	**Females PYAR**	**HIV Cases**	**Incidence**	**R.R**
18–24	98	195	1	5.1	36	68	0	0	
25–29	105	214	4	18.7	26	46	2	43.5	2.3
30–34	51	114	1	8.8	18	35	0	0	
35+	329	727	5	6.9	63	139	0	0	
Total	583	1250	11	8.8	143	288	2	6.9	0.8

*R.R* Relative risk.

*PYAR* Person years at risk.

The age group with the highest HIV incidence for males and females was 25–29 years with incidence rates of 18.7 and 43.5 per 1000 PYAR for males and females respectively.

## Discussion

This study has found out that the overall HIV prevalence and incidence among the police officers in Dar es Salaam has decreased to 5.2% and 8.5 per 1000 PYAR respectively over the past decade as compared to the previous study [7]. In 1994/96, the prevalence and incidence of HIV among the police were 13.8% and 19.9 per 1000 PYAR respectively [7]. The prevalence of HIV among the police is comparable to the national figures in the general population of Tanzania of 6% [3].

We observed that HIV was more prevalent among the married compared to the never married and HIV was also more prevalent among those with lower levels of education compared to those with higher levels of education. This finding is in-keeping with a previous study in Tanzania [11].

We also found that the HIV incidence was high among the age group of 25–29years for both males and females. When comparing this to the previous study that was conducted in 1994/96 the HIV incidence for females in the same age group has doubled from 20.4 per 1000 to 43 per 1000 in the present study. For males no such difference was observed, i.e. 17.1 per 1000 to 19 per 1000 in the present study. However we noted that the higher HIV incidence among males and females occurred in a much younger age group of 25–29 in this study compared to 30–34 years in the 1994/96 study [7].

The reduction in the HIV prevalence and incidence among the police could be attributed to the continuous HIV educational sessions that have been ongoing on average at weekly intervals in the police force. The study team had recruited the police for a phase 1/II HIV vaccine trial and prior to recruitment in the study, continuous HIV prevention educational sessions have been conducted in the various stations in Dar es Salaam. Furthermore there has been massive campaigns country- wide against HIV and AIDS, which might have also resulted into the decrease in prevalence country wide as well as in the police force.

It was also learnt that at the beginning of 2005 there was a policy for mandatory HIV screening of new police recruits and only those found to be HIV-uninfected were recruited into the police force. We speculate that this factor might have also contributed to the low prevalence noted in the current study. Perhaps the HIV infected screened out recruits from the police force could have increased the prevalence in this study. Furthermore the low incidence observed in our study could also be attributed to the fact that the current study might have selected for police who might have been at low risk, as almost 30% of the police who participated in the prevalence study did not re-test in the incidence study.

Generally there has been a reported decline in HIV prevalence and incidence in Tanzania in the general population that is comparable to our study [2].

On comparing these study findings to the previous incidence study conducted among the police in 1996 [7] whereby 2,733 police we recruited over a 3-year period, we only recruited 1240 police over a similar interval of time. We speculate that the low recruitment over time could have been attributed to the availability of HIV testing facilities in the city including a testing center at the police health center.

Of recent there has been an increase in voluntary counseling and testing centers in Tanzania. We noted that some of the police had tested for HIV in less than 3 months at these centers and knew their statuses. Some of the police when traced could not be followed up as they were assigned special duties outside the city or abroad.

During the incidence study we changed to rapid testing for HIV whereby we tested and offered results on site on the same setting. In addition we also offered other tests such as hemoglobin, glucose and urine examination so as to motivate the police to test. However we might have selected for police who had tested themselves prior and had known that they were uninfected and therefore participated in this study resulting to the underestimation of the prevalence and incidence.

For HIV efficacy trials there is a need to recruit a high-risk cohort so as to study the effect of the vaccine in the midst of other existing preventive measures and to reach to a primary endpoint in a relatively short period of time. The incidence of 8.5 per 1000 PYAR observed in the current study clearly indicates that the population studied was a low risk cohort and therefore might not be a suitable cohort for HIV vaccine efficacy studies though a decade ago the cohort appeared to be suitable for efficacy trials. However, there might be several factors that motivated individuals to participate in vaccine trials, one of the factors could be self-perception of being at high risk. In the recently concluded phase I/II HIV vaccine study (HIVIS03 study) that was conducted among the police in Dar es Salaam over a two year period 2/60 recruited volunteers acquired HIV infection during the study this amounts to an incidence of 16.7 per 1000 PYAR [8]. These police who participated in the vaccine trial are from the same stations where the incidence study was conducted and yet the incidence noted in our study was half of that noted in the vaccine study. This might therefore indicate that the police who came to test in the incidence study might have perceived themselves to be at low risk and therefore our study selected for individuals at low risk.

## Conclusions

There has been an overall decline in the HIV prevalence and incidence in the police cohort. Therefore the police in Dar es Salaam are a suitable cohort for phase I/II HIV vaccine studies but might not be a suitable cohort for efficacy studies. Hence efforts are continued to explore other possible cohorts such as young females at high risk for HIV in Dar es Salaam to participate in future efficacy trials.

## Competing interests

The authors declare that they have no competing interests.

## Authors’ contributions

PJM was responsible for the design, data acquisition, analysis and interpretation and drafting the manuscript. MB, BH and ES participated in study design and provided supervision towards data analysis, interpretation and critically revising the manuscript. MJ, EA participated in the design, data collection and revising of the manuscript. SA participated in processing the samples and revising the manuscript. All authors read and approved the final manuscript.

## Pre-publication history

The pre-publication history for this paper can be accessed here:

http://www.biomedcentral.com/1471-2458/13/722/prepub

## Support

Financial support: Swedish International Development Cooperation Agency (SIDA), Department for Research Cooperation (SAREC).
